# Life before and after COVID-19: The ‘New Normal’ Benefits the Regularity of Daily Sleep and Eating Routines among College Students

**DOI:** 10.3390/nu14020351

**Published:** 2022-01-14

**Authors:** Catalina Ramírez-Contreras, María Fernanda Zerón-Rugerio, Maria Izquierdo-Pulido

**Affiliations:** 1 Department of Nutrition, Food Science, and Gastronomy, Food Science Torribera Campus, University of Barcelona, 08921 Barcelona, Spain; catalinaramirez.nut@gmail.com (C.R.-C.); fernanda.zeron@ub.edu (M.F.Z.-R.); 2 Nutrition and Food Safety Research Institute, INSA-UB, 08921 Barcelona, Spain

**Keywords:** daily routines, social jet lag, eating jet lag, COVID-19, sleep, meal timing

## Abstract

After the COVID-19 lockdown, a ‘new normal’ was established, involving a hybrid lifestyle that combined face-to-face with virtual activity. We investigated, in a case-control study, the impact of the ‘new normal’ on daily sleep and eating routines, compared with pre-pandemic conditions. To do this, we propose using social and eating jet lag as markers of the regularity in daily routines. Additionally, we studied whether the ‘new normal’ had an impact on the body mass index (BMI), diet quality, and other health-related variables. This study included 71 subjects in the pre-pandemic group, and 68 in the ‘new normal’ group (20–30 years). For all participants, we evaluated social and eating jet lag, BMI, diet and sleep quality, eating behaviors, physical activity, and well-being. General linear models were used to compare outcome variables between pre-pandemic and ‘new normal’ groups. The results revealed that the ‘new normal’ was associated with greater regularity in daily sleep and eating routines (−0.7 h of social jet lag (95% CI: −1.0, −0.4), and −0.3 h of eating jet lag (95% CI: −0.5, −0.1)), longer sleep duration on weekdays (1.8 h (95% CI: 1.5, 2.2)), and lower sleep debt (−1.3 h (95% CI: −1.7, −0.9)). Regarding BMI and other health-related variables, we observed that these variables were similar between ‘new normal’ and pre-pandemic groups. These findings indicate that the ‘new normal’ had a positive impact on daily sleep and eating routines. Additionally, our results indicated that the ‘new normal’ offered college students a more sustainable lifestyle, which was associated with more hours of sleep during the week and lower sleep debt. This, in the long run, could have a positive impact on BMI and overall health.

## 1. Introduction

In modern societies, infrastructure development and urbanization have positively influenced people’s lifestyles by allowing greater access to food and education [1]. However, these factors can also work in the opposite direction through exposure to artificial light at night, long working hours, longer commutes, and increased general stress associated with living in urban areas [1,2]. Not surprisingly, evidence has shown that the modern lifestyle is associated with obesity, unhealthy eating patterns, and a sedentary lifestyle [1,3]. Furthermore, the modern lifestyle can also have an adverse impact on our daily sleeping and eating routines, which include “when” we sleep, and “when” we eat. Note that access to artificial light at night has allowed us to eat and stay awake at almost any time of the day, associating the latter with late sleep onset, short sleep duration, and also with a mismatch between internal circadian rhythms and external time (known as circadian misalignment) [2,4,5].

In the general population, the most extreme example of circadian misalignment is seen among shift-workers. However, college students, who usually push activities to a later clock time, are prone to a mild type of circadian misalignment, denominated social jet lag [4,5,6,7,8]. The latter arises from the accumulated sleep debt throughout the week, especially since school schedules tend to start early, which forces them to align their waking hours with the social obligations of the weekdays [5,7]. Nevertheless, since sleep debt is unsustainable, young people generally prolong sleep duration on weekends, resulting in a discrepancy in their sleep routines on weekends versus weekdays [6,9]. Note that social jet lag is considered a potential risk factor for obesity [6,7,10,11] and unhealthy eating habits among college students [6,11]. In this context, insufficient sleep is one of the main mechanisms that trigger these associations [7]. In particular, the short duration of sleep is related to a higher food intake [12], mainly due to alterations in appetite hormones (e.g., ghrelin and leptin), hedonic eating, and a longer time awake, which translates into more opportunities to eat and snack [12,13,14].

Furthermore, it has been reported that individuals with the greatest social jet lag are also those who eat breakfast and dinner later [11]. Our group has demonstrated that irregularity in the timing of meals throughout the week, especially at breakfast time, is significantly associated with a greater social jet lag [9]. Due to its resemblance with social jet lag, we denominated this irregularity in eating routines as ‘eating jet lag’. In this regard, we have shown that a greater eating jet lag was significantly associated with a higher body mass index (BMI) among college students. Similar to the circadian desynchrony that arises from social jet lag, we hypothesized that eating jet lag could be linked to circadian misalignment, and therefore has a negative impact on BMI [9]. It should also be noted that a greater ‘jet lag’ at the time of the first meal has also been associated with a higher BMI and waist circumference among women [15].

In light of the evidence, it seems reasonable to recommend maintaining regular sleep and eating routines throughout the week, which would presumably be associated with a lower BMI and healthier eating habits. However, due to the modern lifestyle, this seemed impossible. It was not until the social restrictions issued in response to the COVID-19 pandemic showed that a greater flexibility in social schedules and less time spend commuting had positive changes in terms of sleep routines [2,16,17,18]. Specifically, studies showed that lockdown was associated with later wakeup time, longer sleep duration, and lower social jet lag [2,16,17,18]. However, the benefits of regular daily sleep and eating routines on BMI and diet quality remain to be studied. To our knowledge, only Blume et al. [17] found that regular sleep routines (given by lower social jet lag) limited the decline in sleep quality and well-being during lockdown.

In our country, after the strict lockdown period, economic and social activities resumed at the end of June 2020. Therefore, a ‘new normal’ was established, which included, among others, a hybrid lifestyle that combined face-to-face with virtual activity [19]. Implicitly, the need to resume life after COVID-19 lockdown through the ‘new normal’ meant that, in relation to pre-pandemic conditions, there would be a before and after in terms of daily sleeping and eating routines, and their regularity. Therefore, the objective of our research was to evaluate the impact of the ‘new normal’ on daily sleep and eating routines in relation to pre-pandemic conditions. To do this, we propose using social and eating jet lag as markers of regularity in daily sleep and eating routines. We hypothesized that, compared with pre-pandemic conditions, the ‘new normal’ would be associated with lower social and eating jet lag, and thus, greater regularity in sleep and eating routines. In addition, we analyzed whether the ‘new normal’ had an impact in BMI, diet quality, and other health-related variables (including eating behaviors, sleep quality, physical activity, and well-being).

## 2. Materials and Methods

### 2.1. Study Design and Participants

Undergraduate students (aged 20–30 years) of the Bachelor’s Degree in Human Nutrition and Dietetics at the University of Barcelona (Barcelona, Spain) were recruited in November 2019 (pre-pandemic) and November 2020 (‘new normal’) for a case-control study. Note that in Catalonia (Spain), the ‘new normal’ included the mandatory use of face-masks, social distancing, and a hybrid lifestyle that combined face-to-face with virtual activity. Additionally, starting in October 2020, this ‘new normal’ also included a curfew from 22:00 to 6:00, prioritizing the home-office when possible, and, at university, classes were taught online [19]. Social life and leisure time were allowed (at least in gatherings of fewer than six people), restaurants and cultural activities were opened from 6:00 to 21:30, and individual outdoor exercise was allowed [19].

### 2.2. Recruitment

Recruitment consisted of an informative talk, in which the details of the research were explained to the students, and they were invited to participate in the study. The eligibility criteria included college students enrolled in the University of Barcelona aged between 20 and 30 years old. Exclusion criteria consisted of unwillingness to participate in the study, and the participants who provided incomplete information required for the development of the study. Based on these criteria, a total of 150 subjects were included in the study, all of whom gave written informed consent. Furthermore, we excluded subjects with missing information (*n =* 11), which resulted in a final analytical sample of 139 subjects. All study procedures were performed according to the ethical guidelines of the Declaration of Human Studies of Helsinki, and were approved by the Ethics Committee of the University of Barcelona (IRB00003099).

### 2.3. Data Collection

We used Open Data Kit (ODK) [20], which is an open-source software, to design an online screening tool, where we included a series of validated questionnaires (detailed below) to evaluate the chronotype, diet quality, eating behaviors, physical activity, and well-being. ODK has a user-friendly web interface for designing web forms and programming simple logic.

### 2.4. Markers of Daily Routines

#### 2.4.1. Sleep Routines

Participants completed a sleep diary during seven consecutive days, where they recorded bedtimes and wakeup times. From these data, we calculated the following variables:

i. Sleep duration (h), calculated as the difference between bedtime and wakeup time.
ii. Social jet lag (h), calculated as the difference between each participant’s midpoint of sleep on weekdays and midpoint of sleep on weekends [5]. All analyses were performed using the absolute value of social jet lag [5].
iii. Sleep debt (h), calculated as the difference in sleep duration between weekends and weekdays [21].

#### 2.4.2. Eating Routines

Participants completed a meal timing diary during seven consecutive days, where they reported the timing of the meals (breakfast, lunch, dinner, or any other meal). From these data, we calculated the following variables:

i. Eating duration (h), calculated as the length between the first and the last caloric event [11].
ii. Eating jet lag (h), calculated as the difference between each participant’s eating midpoint on weekends and eating midpoint on weekdays [9]. All analyses were conducted using the absolute value of eating jet lag.

### 2.5. Anthropometric Parameters

Weight was measured with a body composition analyzer (InBody 720, Biospace, Seoul, Korea), with the subjects wearing light clothing and without shoes, to the nearest 0.1 kg. Height was determined using a fixed wall stadiometer (Seca 217, Seca, Hamburg, Germany) to the nearest 0.1 cm. Body mass index (BMI) was calculated as weight (kg) divided by height squared (m).

### 2.6. Health-Related Variables

#### 2.6.1. Diet Quality

Diet quality was evaluated using the Mediterranean Diet Quality Index (KIDMED), which has been validated in the Spanish population [22]. The KIDMED test is based on the principles that underpin Mediterranean dietary patterns and those that undermine it. Briefly, the KIDMED test includes questions such as: ‘Do you have fruit or fruit juice every day?’, ‘Do you have fresh or cooked vegetables regularly once a day?’, ‘Do you consume nuts regularly (at least 2–3 times per week)?’, and ‘Do you go more than once a week to a fast-food (hamburger, pizza) restaurant?*’*. Items denoting lower adherence to the Mediterranean diet were assigned a value of −1, and those related to higher adherence were scored +1. KIDMED scores range from −4 to 12 points, where the higher the score, the better the diet quality.

#### 2.6.2. Eating Behaviors

Eating behaviors were assessed using the Three Factor Eating Questionnaire (TFEQ-R21C) [23]. Briefly, the TFEQ-R21C includes 21 items such as: ‘I’m always hungry enough to eat at any time’, ‘I start to eat when I feel anxious’, ‘I take small portions on purpose to control my weight’, ‘When I feel lonely, I console myself by eating’, ‘I don’t eat some food because they make me fat’. These items are used to evaluate the following dimensions of eating behavior:

i. Cognitive restraint, understood as the conscious efforts of individuals to control what they eat to maintain or lose weight.
ii. Uncontrolled eating, which expresses the tendency to eat excessively in response to the loss of control over the food itself.
iii. Emotional eating, understood as the need to overeat when individuals are unable to cope with emotionally negative situations and moods.

The TFEQ-R21C consists of 21 items that are scored on a four-point Likert scale ranging from 1 (‘Definitely true’) to 4 (‘Definitely false’). Scores are calculated separately for each dimension as a mean of all items, where the higher the score, the greater the emotional eating, the cognitive restraint, and/or the uncontrolled eating [23,24].

#### 2.6.3. Physical Activity

The level of physical activity was evaluated using the short version of the International Physical Activity Questionnaire (IPAQ) [25]. This version of the IPAQ questionnaire has been validated in the Spanish population, in which a good correlation with accelerometer data was obtained. The IPAQ contains questions such as: ‘During the last 7 days, on how many days did you do vigorous physical activities like heavy lifting, digging, aerobics or fast bicycling?’, ‘How much time did you usually spend doing vigorous physical activities on one of those days’, ‘During the last 7 days, on how many days did you do moderate physical activities like carrying light loads, bicycling at a regular pace, or doubles tennis? Do not include walking’, ‘How much time did you usually spend doing moderate physical activities on one of those days’, ‘During the last 7 days, on how many days did you walk for at least 10 minutes at a time?’, and ‘How much time did you usually spend walking on one of those days?’. The physical activity score was calculated in Metabolic Equivalents of Task (MET)-minutes per week. In this case, the higher the score, the more intense the level of physical activity.

#### 2.6.4. Sleep Quality

Additionally, sleep quality was evaluated with the Pittsburg Sleep Quality Index (PSQI) [26], which has been validated in Spanish population [27]. The PSQI contains questions such as: ‘during the past month, how often have you had trouble sleeping because you had bad dreams?’, ‘during the past month, how would you rate your sleep quality overall?’, ‘during the past month, how often have you had trouble staying awake while driving, eating meals, or engaging in social activities?’. The PSQI consists of 19 items, each rated on a four-point scale (0–3), grouped into seven components: subjective sleep quality, sleep latency, sleep duration, habitual sleep efficiency, sleep disturbance, use of sleeping medications, and daytime dysfunction. Scores range from 0 to 21, where the higher the score, the worse the sleep quality.

#### 2.6.5. Well-Being

This variable was assessed using the World Health Organization-5 (WHO-5) Well-Being Index [28]. The WHO-5 includes five statements: ‘I have felt cheerful and in good spirits’, ‘I have felt calm and relaxed’, ‘I have felt active and vigorous’, ‘I woke up feeling fresh and rested’ and ‘My daily life has been filled with things that interest me’. The respondent is asked to rate, on a five-point Likert scale (from 0 ‘at no time’ to 5 ‘all the time’), how well each of the five statements apply to him or her when considering the last 14 days. The total raw score is multiplied by 4 to obtain a final score on the scale from 0 to 100, where the higher the score, the higher the well-being.

### 2.7. Statistical Analyses

Normality was confirmed through histograms and Q–Q plots. Descriptive characteristics are presented for all participants, including mean and standard deviation for continuous variables, and proportions for categorical variables. A chi-squared test was used to compare gender between pre-pandemic and ‘new normal’ groups. Meanwhile, General Linear Models (GLMs) were used to compare age, and sleep and eating routines between pre-pandemic and ‘new normal’ groups. In addition, we used GLMs to calculate adjusted differences in variables related to sleep and eating routines (reference category ‘pre-pandemic’). Subsequently, we compared BMI, diet quality, eating behaviors, physical activity, and well-being variables between pre-pandemic and ‘new normal’ groups using GLMs. All *p*-values were corrected using the Benjamini–Hochberg method, assuming a False Discovery Rate (FDR) of 5%. All analyses were adjusted for age and gender, and were performed with the SPSS statistical computer software, version 25.0 (IBM SPSS Statistics, Armonk, NY, USA). Significance testing was considered when *p <* 0.05.

## 3. Results

A total of 139 college students were included in this case-control study. The mean age of the participants in the pre-pandemic group (*n =* 71) was 22.5 ± 2.3 years, whereas the subjects in the ‘new-normal’ group (*n =* 68) were 22.8 ± 3.1 years old (*p =* 0.446). Regarding gender, most of the participants were women (pre-pandemic 81.7%, and ‘new normal’ 88.2%), with no statistically significant differences between groups (*p =* 0.281).

**Figure 1:**
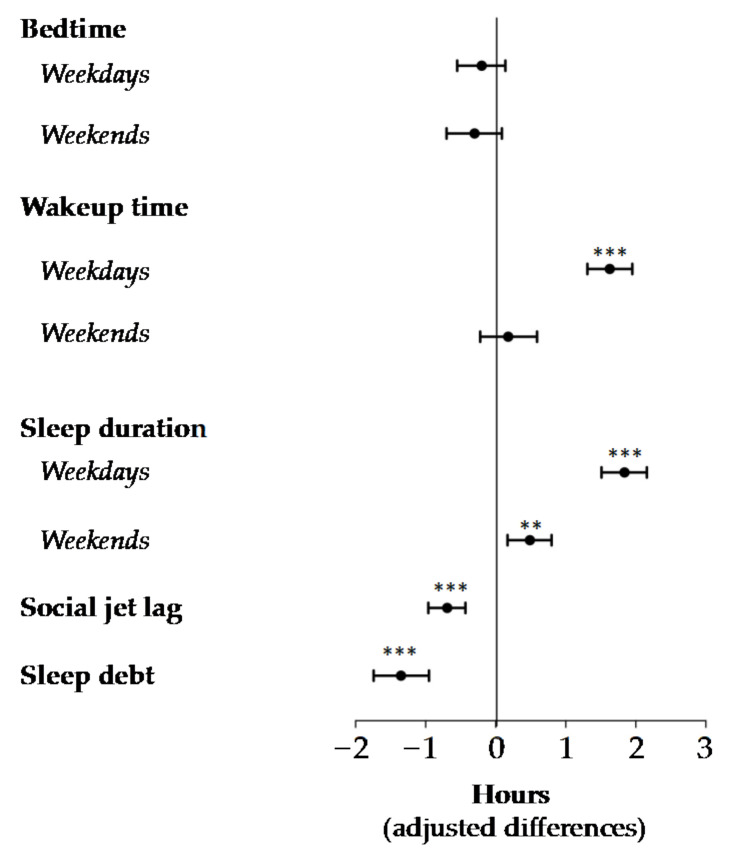
Changes in sleep routines between ‘new normal’ and pre-pandemic groups. General linear models were used to calculate adjusted differences in sleep routines between ‘new normal’ and pre-pandemic conditions (reference group “pre-pandemic”). Analyses were adjusted for age and gender. *p*-values were corrected using the Benjamini–Hochberg method, assuming a False Discovery Rate (FDR) of 5%. ** *p <* 0.01, *** *p <* 0.001.

Regarding sleep routines, our results revealed that though bedtime was similar between pre-pandemic and ‘new normal’ groups, wakeup time differed significantly on weekdays (06:44 ± 00:59 vs. 08:19 ± 00:57, *p <* 0.001). Specifically, we observed that during the ‘new normal’, participants woke up 1.6 h (95% CI: 1.3, 1.9) later on weekdays (Figure 1). In addition, we observed a significant increase in sleep duration on both weekdays and weekends during the ‘new normal’. However, the greatest increase was found on weekdays, when sleep duration was 1.8 h longer (95% CI: 1.5, 2.2) (Figure 1).

Interestingly, our results showed a significant reduction in social jet lag and sleep debt during the ‘new-normal’ compared to pre-pandemic conditions. In this regard, we noted that the participants reduced their social jet lag to almost one hour (−0.7 h (95% CI: −1.0, −0.4)), whereas sleep debt was reduced by 1.3 h (95% CI: −1.7, −0.9) (Figure 1).

As for eating routines (Table 1), we observed that eating jet lag was significantly reduced in the ‘new normal’ (−0.3 h (95% CI: −0.5, −0.1)). We also found a slight advance in dinner time during weekdays (*p <* 0.05), in which case, dinner was 0.2 h earlier (95% CI: −0.44, −0.04) in the ‘new normal’. In addition, and despite the significant difference in wakeup time on weekdays, we observed that breakfast time on weekdays was similar between the pre-pandemic and ‘new normal’ groups (Table 1).

Our results also indicated that BMI and diet quality were similar between the pre-pandemic and ‘new normal’ groups (Table 2). Regarding eating behaviors, the results showed that cognitive restraint was reduced by 0.2 points (95% CI: −0.4, −0.1) in the ‘new normal’, whereas no differences were found in emotional and uncontrolled eating scores. Regarding sleep quality and physical activity, we did not observe any significant difference between groups (Table 2). Likewise, we observed that well-being was similar between the pre-pandemic and ‘new normal’ groups.

## 4. Discussion

Our findings revealed that, relative to pre-pandemic conditions, the ‘new normal’ had a positive impact in terms of regularity in daily sleep and eating routines. Therefore, the greater flexibility in social schedules provided by the ‘new normal’ was significantly associated with lower social and eating jet lag (−0.7 h and −0.3 h, respectively). Furthermore, our results suggest that in the ‘new normal’, the sleep routines followed by college students on weekdays were more sustainable. Note that in the ‘new normal’, participants slept 1.8 h more, and sleep debt was reduced by ~1.3 h.

These findings are in line with the conclusions drawn from COVID-19 lockdown studies indicating that greater flexibility in social schedules, possibly due to online learning and the elimination of commute time, had a positive impact on daily sleep routines [2]. This is supported by the significant decrease in sleep debt and social jet lag found during the ‘new normal’. Furthermore, our results showed that the ‘new normal’ lifestyle could mitigate the misalignment between biological and social clocks [2,16,17,18]. It should be noted that circadian misalignment has been associated with obesity and metabolic alterations [6,7,10,11], as well as with unhealthy eating habits [6,29]. Therefore, it is plausible that, in the long term, regularity in daily sleep routines could have a positive impact on body weight and other health-related variables in college students. However, evidence from longitudinal studies needs to be warranted.

Regarding eating routines, we noticed a slight advance in dinner timing on weekdays (~0.24 h). Note that advancing dinner time would allow postprandial blood glucose to return to fasting values before the rise in endogenous melatonin levels [30]. In fact, having dinner closer to bedtime is associated with obesity and metabolic alterations [4,30,31]. Furthermore, this subtle shift in dinner timing plus the regularity in daily sleep routines seen in the ‘new normal’ are related to a lower eating jet lag. Regularity in daily eating routines is crucial to maintaining optimal nutrient utilization [32,33]. Note that, when eating occurs at an expected (or regular) time, the circadian system ensures that the proper pathways that help to assimilate the nutrients begin to increase in anticipation of food intake [32]. However, when food intake occurs at an unexpected (or irregular) time, nutrient sensing pathways act on the peripheral clocks so that food is anticipated at the new mealtime in the following days [32]. Thus, eating can independently activate nutrient-sensing pathways, compromising the way food is processed during the postprandial period. Not surprisingly, the irregularity in daily eating routines (given by a greater eating jet lag) has been associated with obesity [9,15].

It is worth noting that regularity in daily sleep and eating routines might explain why BMI was similar between the ‘new normal’ and the pre-pandemic groups. We cannot ignore that the COVID-19 lockdown was a stressful time that, among others, had a negative impact on what we ate, how well we slept, and how much exercise we practiced [17,34,35,36]. Unsurprisingly, during the COVID-19 lockdown, people were more likely to gain weight. In fact, a recent systematic review and meta-analysis found that body weight and BMI increased significantly (~1.57 kg and ~0.31 kg/m^2^, respectively) during the lockdown period compared with pre-pandemic conditions [37]. Thus, our results suggest that the measures that characterized the ‘new-normal’ (such as prioritizing online classes, allowing individual exercise, and letting people have some sort of social life) may have helped college students to maintain and/or recover their weight once they returned to ‘normal’ life.

In line with the above, we observed that diet quality was similar between the ‘new normal’ and pre-pandemic groups. Our hypothesis was that schedule flexibility could play a role in maintaining diet quality, as having online classes could give college students more time to do other activities, such as cooking. This trend was also observed in young Spanish adults during the COVID-19 lockdown [34]. The authors reported that 57% of the population studied increased their home cooking practices [34], which could favor the consumption of healthier homemade foods [38]. It is worth mentioning that meals prepared and eaten at home are associated with higher-quality diets and better health outcomes [39,40,41]. Specifically, Larson et al. [39] showed that young adults who frequently bought their own food and prepared meals at home had a better diet quality. Interestingly, adherence to a healthy diet could play a significant role in the prevention and predisposition to viral infections, such as COVID-19 [42,43]. According to recent reviews, special attention should be paid to nutrients that play a role in regulating the immune response [43]. For example, Messina et al. [42] hypothesized that omega-3 polyunsaturated fatty acids could be used to reduce inflammation, as well as to ameliorate lung damage that occurs after coronavirus infection.

It is also worth noting that in the ‘new normal’, college students were more relaxed in terms of their diet, without compromising diet quality. It is noteworthy that our results showed that cognitive restraint was significantly lower in the ‘new normal’ compared with the pre-pandemic group. Importantly, less cognitive restraint does not necessarily imply that the subjects are prone to increase their body weight, but quite the opposite [44,45]. In fact, previous research performed by our group showed that a greater dietary restraint is associated with a higher BMI among college students [45].

Along these lines, we observed that the flexibility provided by the ‘new normal’ could have helped to maintain sleep quality, despite the stressful pandemic context. This would also be in line with the findings of Blume et al. [17] during COVID-19 lockdown. According to the authors, the reduction in sleep debt and social jet lag limited the decline in sleep quality during lockdown. Furthermore, Pilz et al. [46] suggested that it is not the delay in sleep timing that affects sleep quality, but rather the social jet lag. The authors explained that the combination of late sleep schedules with the time constrains of the social clock could explain why subjects who had a preference for late sleep schedules usually showed the worst sleep quality.

Regarding other health-related variables, our results revealed that physical activity was similar between the ‘new normal’ and pre-pandemic groups (~2242.8 vs. ~2193.9 MET-minutes/day, respectively). This can be attributed to the fact that, despite the restrictions, in the ‘new normal’, exercise was allowed. Importantly, Zhang et al. [47] observed that during COVID-19 lockdown, performing 2500 METs/week of physical activity (equivalent to a moderate level of physical activity [48]) alleviated negative emotions in college students, which could also be in line with our results regarding well-being. It is worth noting that despite social restrictions and the curfew issued in the ‘new normal’, well-being remained similar between the ‘new normal’ and pre-pandemic groups. It is also plausible that the consistency of daily sleep routines could also be associated with the maintenance of well-being in the ‘new normal’ [2,49].

Our study has certain limitations, starting with the observational nature of the study, that prevent us from claiming causation. Additionally, we acknowledge as a limitation that our sample consisted mostly of women and undergraduate students of the Bachelor’s Degree in Human Nutrition and Dietetics, which is not representative of the entire population. Furthermore, the representativeness of our results is limited to students living in an urban area. We also acknowledge our results are based upon a cohort of healthy young adults, who may not be representative of the entire population in terms of sleep and meal timing. Nonetheless, the strength of our study is that this is the first research to study the effects of the ‘new normal’ on daily sleep and eating routines, BMI, and health-related variables in college students.

## 5. Conclusions

In summary, our findings indicate that the ‘new normal’ was associated with greater regularity in daily sleeping and eating routines. Additionally, we observed that BMI, diet and sleep quality, the level of physical activity, and well-being were similar between the ‘new normal’ and pre-pandemic groups. Although, we did observe that college students were less restrictive in terms of their diet, without compromising diet quality. It is also worth noting that the ‘new normal’ offered college students a more sustainable lifestyle, which was reflected in longer sleep duration on weekdays, as well as with less social and eating jet lag. These findings point to the need to rethink the possibility of combining face-to-face activities with remote work and online education, which could be associated with more hours of sleep during weekdays, and the regularity of daily sleep and eating routines. However, more studies are needed to investigate the long-term potential benefits of regular sleep and eating routines on BMI and other health-related variables. Finally, our findings could help formulate public health recommendations for future pandemics where social distancing measures are needed to halt the spread of a virus.

## Figures and Tables

**Table 1:** Comparison of eating routines between pre-pandemic and ‘new normal’ groups.

	Pre-Pandemic (*n =* 71)	New Normal (*n =* 68)	*p*-Value ^a^
Eating jet lag, h	0.9 (0.7)	0.6 (0.5)	**0.022**
Breakfast			
Weekdays, hh:mm	09:10 (01:22)	09:05 (00:58)	0.141
Weekends, hh:mm	10:11 (01:22)	09:52 (01:00)	0.284
Lunch			
Weekdays, hh:mm	14:02 (00:39)	14:10 (00:30)	0.383
Weekends, hh:mm	14:30 (01:57)	14:28 (00:34)	0.494
Dinner			
Weekdays, hh:mm	21:37 (00:41)	21:22 (00:28)	**0.045**
Weekends, hh:mm	21:32 (00:57)	21:31 (00:39)	0.542
Eating duration			
Weekdays, hh:mm	12.4 (1.5)	12.1 (1.1)	0.892
Weekends, hh:mm	11.0 (1.8)	11.5 (1.0)	0.176

Values are mean and standard deviation (SD) for continuous data. ^a^ Statistical analyses: general linear models were used to compare eating routines between pre-pandemic and ‘new normal’ groups. Analyses were adjusted for age and gender. *p*-values were corrected using the Benjamini–Hochberg method, assuming a False Discovery Rate (FDR) of 5%. Significant *p*-values are shown in bold.

**Table 2:** Comparison of pre-pandemic and ‘new normal’ conditions in body mass index and health-related variables.

	Pre-Pandemic (*n =* 71)	New Normal (*n =* 68)	*p*-Value ^a^
Body mass index, kg/m^2^	22.2 (3.2)	21.3 (2.7)	0.177
Diet quality, score	8.9 (1.8)	8.5 (1.9)	0.366
Eating behaviors			
Cognitive restraint, score	2.1 (0.5)	1.9 (0.3)	**0.027**
Emotional eating, score	1.7 (0.6)	1.7 (0.6)	0.893
Uncontrolled eating, score	1.9 (0.5)	1.9 (0.4)	0.707
Sleep quality, score	5.1 (2.4)	5.2 (2.5)	0.818
Physical activity, MET-minutes/day	2242.8 (1591.1)	2193.9 (1913.7)	0.899
Well-being, score	57.8 (16.6)	55.2 (17.6)	0.550

Values are mean and standard deviation (SD). MET, metabolic equivalent of task. **^a^** Statistical analyses: general linear models were used to compare body mass index and health-related variables between pre-pandemic and ‘new normal’ groups. Analyses were adjusted for age and gender. *p*-values were corrected using the Benjamini–Hochberg method, assuming a False Discovery Rate (FDR) of 5%. Significant *p*-values are shown in bold.
